# Development and Validation of a Novel Nomogram to Predict the Risk of Intervertebral Disc Degeneration

**DOI:** 10.1155/2022/3665934

**Published:** 2022-09-10

**Authors:** Fudong Li, Xiaofei Sun, Yuan Wang, Lu Gao, Jiangang Shi, Kaiqiang Sun

**Affiliations:** ^1^Department of Orthopedic Surgery, Shanghai Changzheng Hospital, Navy Medical University, Shanghai 200003, China; ^2^Department of Physiology, Navy Medical University, Shanghai 200433, China; ^3^Department of Orthopaedic Surgery, Naval Medical Center, Navy Medical University, Shanghai 200433, China

## Abstract

Intervertebral disc degeneration (IVDD) has been a complex disorder resulted from genetic and environmental risk factors. The aim of this study was to identify the risk factors associated with IVDD in orthopaedic patients and develop a prediction model for predicting the risk of IVDD. A total of 309 patients were retrospectively included in the study and randomly divided into the training group and the validation group. The least absolute shrinkage and selection operator regression (LASSO) and the univariate logistic regression analysis were used to optimize factors selection for the IVDD risk model. Multivariable logistic regression analysis was used to establish a predicting nomogram model incorporating the factors. In addition, discrimination, calibration, and clinical usefulness of the nomogram model were evaluated via the C-index, receiver operating characteristic (ROC) curve, calibration plot, and decision curve analysis (DCA). Then, based on the results above, the relationship between IVDD and angiotensin II (AngII) level in peripheral blood was examined prospectively. The predictors of the nomogram include age, sex, hypertension, diabetes, gout, working posture, and exercising hours per week. The *C*-index values of the training and validation groups were 0.916 (95% CI, 0.876-0.956) and 0.949 (95% CI, 0.909-0.989), respectively, which indicated that the model displayed good discrimination. In addition, the area under the curve (AUC) values of the ROC curve of the training and the validation group were 0.815 (95% CI, 0.759-0.870) and 0.805 (95% CI, 0.718-0.892), respectively, revealing the satisfactory discrimination performance of the model. The prospective investigation showed that the average AngII level in the degenerated group (97.62 ± 44.02 pg/mL) was significantly higher than that in the nondegenerated group (52.91 ± 9.01 pg/mL) (*p* < 0.001). This present study explored the risk factors for IVDD and established a prediction model, which would effectively predict the risk of IVDD. In addition, based on the prediction model, AngII was revealed to be a potentially auxiliary clinical diagnostic marker for IVDD.

## 1. Introduction

Low back pain (LBP) is one of the major causes of disability worldwide, which often leads to bad quality of life. It was estimated that up to 80% of the individuals experienced LBP at some point during their entire lifetime [1]. In addition, it was reported that the lifetime morbidity of neck-related pain was more than 65% [2]. These conditions above impose a huge socioeconomic burden on the society [3]. LBP and neck-related pain are both symptoms resulted from intervertebral disc degeneration (IVDD). IVDD, an ageing-related disorder, is one of the most common diseases in clinical practice. IVDD is widely recognized as a contributor to spinal degenerative diseases, which is characterized by the loss of nucleus pulposus cells and the degradation of extracellular matrix (ECM).

It is widely recognized that IVDD is caused by both environmental and genetic factors. While genetic factors play a critical role in IVDD, it cannot be ignored that environmental factors including living conditions, lifestyle characteristics, and chronic diseases tend to be closely associated with the risk of disc degeneration [4]. Kuisma et al. reported that Modic change was closely associated with IVDD [5]. In addition, IVDD was associated with metabolic diseases such as diabetes and hyperlipidemia [6, 7]. Our previous study revealed that the degenerated IVD tissue showed excessively activated the tissue renin-angiotensin system (tRAS) that can lead to nucleus pulposus cell (NPC) senescence, apoptosis, oxidative stress, and inflammatory reaction [8]. The activation of renin-angiotensin system (RAS) including both the systemic RAS and the tRAS has long been considered an essential part during the development of hypertension [9]. Therefore, we deduced that hypertension may be a potential risk factor for IVDD. What is more, manual handling and postures of the trunk were associated with IVDD. Yuya et al. reported that exercise can attenuate LBP and result in epigenetic alterations in IVDD [10]. In a cross-sectional case-control study, Elke et al. revealed that long-term physical inactivity was significantly associated with IVDD [11]. And it was reported that pain was alleviated and cell proliferation was promoted by running exercise in a rat model of IVDD [12]. Collectively, it is essential to comprehensively understand the clinical characteristics of patients with IVDD to identify the risk factors, which may provide novel insight into future treatment of IVDD.

Nomograms are graphical statistical models that are designed to integrate significant risk factors in numerous diseases and to predict the probability of certain clinical events [13]. It can integrate multiple risk factors into a reliable risk model and visualize the results. Based on potential risk factors, nomograms are superior to other available decision aids for more accurately predicting outcomes in various diseases [14]. Early identification of risk factors for IVDD can facilitate prevention and early intervention for high-risk populations, thereby reducing the socioeconomic burden and surgery-related complications. However, there is only one report on the application of nomogram for the development of IVDD, and its predictive factors were only restricted to blood lipid-metabolism-related genes [15]. In addition, the limited samples in the GEO series can bring about statistical errors, and the data in their study did not contain necessary clinical information of patients. These aspects decreased the predictive power of the model. Taken together, it is imperative to establish a reliable risk prediction model for IVDD. In this study, orthopaedic patients were selected as a specific population to assess the risk factors and to develop a predictive nomogram model of IVDD. The present study is aimed at establishing a valid and simple predictive model to assess the risk of IVDD via assessing controllable environmental factors.

## 2. Methods

### 2.1. Patients and Data Collection

The clinical and imaging data of patients were derived from Shanghai Changzheng Hospital from January 2019 to September 2020. The study was approved by the Ethics Committee of the Shanghai Changzheng Hospital (approval No. CZ20181113). The inclusion criteria include (a) patients with complete medical records; (b) patients with complete imaging data including x-rays, computed tomography (CT), and magnetic resonance imaging (MRI) of the lumbar spine; and (c) patients over 18 years of age. The exclusion criteria include (a) patients who underwent spine surgery before the admission and (b) patients lack of contacting details. According to the criteria, a total of 309 participants were finally enrolled. Data of patients were collected, which included age, sex, smoking, drinking, BMI, hypertension, diabetes, hyperlipidemia, gout, marital status, working posture, exercising hours per week, education level, Modic changes, osteophytes, Ca^2+^, hemoglobin, the history of lumbar puncture, and lumbar Pfirrmann grades.

### 2.2. Assessment of Intervertebral Disc Degeneration

**Figure 1:**
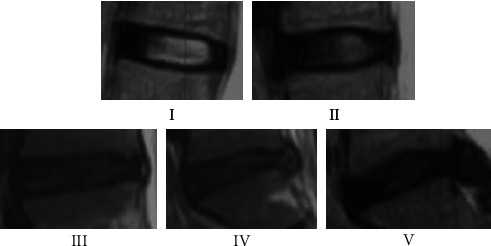
The degree of IVDD is evaluated by the Pfirrmann grade system. The discs with Pfirrmann grades I or II are nondegenerative discs, and the discs with grades III, IV, and V are taken as degenerative discs.

The Pfirrmann grade system was used to evaluate the degree of disc degeneration on the T2-weighted MRI (Figure 1). Details of the Pfirrmann grading system can be found in Reference [16]. Pfirrmann grades I and II were taken as nondegenerative discs, whereas grades III, IV, and V were considered degenerative discs [16]. Based on the Pfirrmann grading system, patients were classified into group N (nondegenerative discs in lumbar) and group D (degenerative discs in lumbar). Two spine surgeons independently evaluated the grades of IVD. If there is disagreement between the two surgeons, it would be discussed and the results were ultimately confirmed by the corresponding author.

### 2.3. Enzyme-Linked Immunosorbent Assay (ELISA)

The samples of peripheral blood from 108 patients with IVDD and of 92 individuals without IVDD were collected between November 2020 and March 2022. The informed consent was signed by the patients. The inclusion criteria of patients with IVDD are as follows: (1) patients with Pfirrmann grades III, IV, and V in the lumbar and cervical spine; (2) patients without hypertension; (3) patients with abnormal motor and sensory function of the upper or lower limbs; (4) patients who agreed to participate in the study; and (5) patients without neuroendocrine dysfunction or adrenal disorders. The inclusion criteria of individuals without IVDD are as follows: (1) individuals without Pfirrmann grades III, IV, and V in the lumbar and cervical spine; (2) individuals without hypertension; and (3) individuals who volunteered to participate in the study. The level of AngII in human peripheral blood was measured using the human Ang-II ELISA Kit (Westang, Shanghai, F00070) according to the manufacturer's instruction.

### 2.4. Statistical Analysis

The demographic and clinical characteristic data in this study were expressed as frequency and percentage. Continuous numeric variables were presented as the mean ± SD. Categorical variables were compared by the *χ*^2^ test or Fisher's exact test. Statistical analysis was carried out with the R software (version R-3.4.3) and SPSS (version 25.0). The data were randomized to the training set and the validation set at the ratio of 7 : 3. The training group was applied to establish the prediction model, and the validation group was used to validate the model. The least absolute shrinkage and selection operator (LASSO) method was used to optimize the features used for multivariate logistic regression analysis. In the LASSO model, variables with nonzero coefficients were selected [17]. Univariate logistic analysis was performed for all the involved independent variables. To avoid missing important factors, we included parameters with *p* < 0.1 into the multivariate analysis [18]. The features selected through the univariate logistic analysis and the LASSO were applied for the multivariate logistic analysis. The independent risk factors of IVDD, which were determined by the multivariate logistic regression analysis, were included to develop a prediction model for IVDD. The nomogram was utilized to visualize the model.

Based on the prediction model, the performance of the nomogram model was assessed in both the training and validation groups. The area under the curve (AUC) of the receiver operating characteristic (ROC) curve and the Harrell's *C*-index were applied to assess prediction power of the model. An AUC of 0.5 implicated no prediction performance; an AUC value ≤ 0.7 indicates poor predictive performance; an AUC value greater than 0.7 but lower than 0.9 indicates moderate predictive performance; and an AUC value greater than 0.9 indicates excellent predictive performance. The *C*-index value of 0.5 suggests that the model is almost random chance in predicting the risk, whereas a value of 1.0 indicates perfect discrimination. The calibration process examines whether the predicted risks and the observed risks are consistent. To assess the clinical utility of the model, decision curve analysis (DCA) was used to evaluate the benefit. *p* < 0.05 was regarded statistically significant.

## 3. Result

### 3.1. Demographic and Clinical Characteristics

Details of the patients are shown in Table 1. A total of 309 participants were included. They were randomly divided into the training group (*n* = 217) and the validation group (*n* = 92). The training group was used to establish the nomogram for predicting the risk of IVDD. The validation group was used for validation. There were 92 (42.40%) and 40 (43.48%) patients with IVDD in the training cohort and the validation cohort, respectively. No significant differences were observed regarding the basic parameters between the two groups.

### 3.2. Selection of Independent Variables

**Figure 2:**
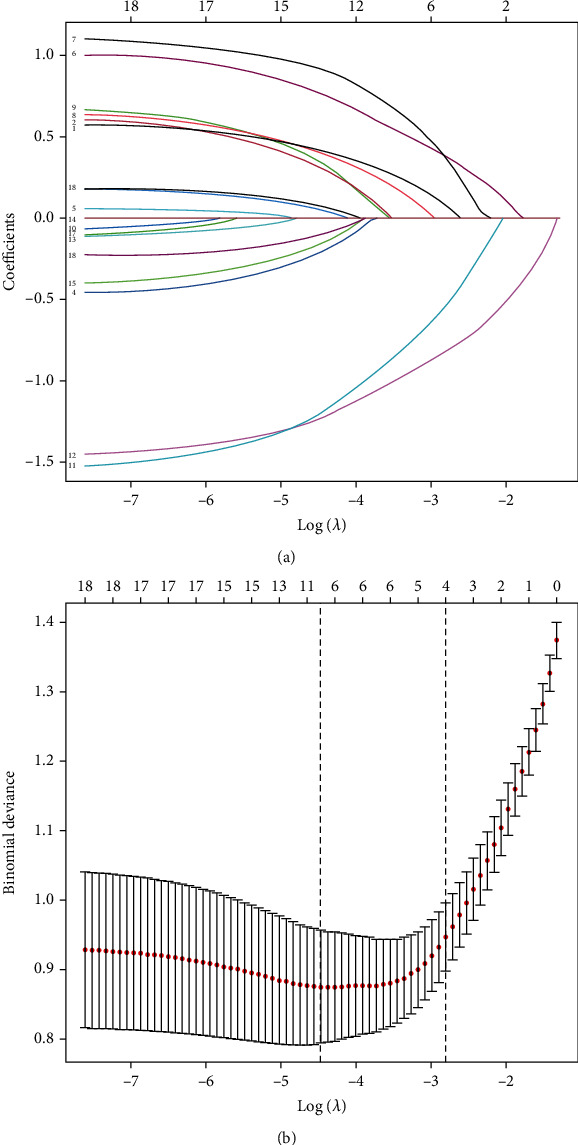
Results of the LASSO regression analysis. (a) LASSO coefficient profiles of the 18 features. (b) Feature selection in the LASSO. Dotted vertical lines were drawn at the optimal values.

IVDD occurred in 92 (42.40%) of the 217 patients in the nomogram development cohort. Univariate logistics regression and LASSO regression analysis were used for the selection of potential predictors. In this present study, parameters with *p* values less than 0.1 in the univariate logistic analysis were regarded as potential predictors for the nomogram model, which included age, smoking, hypertension, diabetes, gout, working posture, exercising hours per week, education level, osteophytes, Ca^2+^, and Hb (Table 2). LASSO regression analysis helped decrease the dimensionality that was associated with the decreased predictive power and increased the accuracy of the nomogram model. Variables in the LASSO regression analysis, which included age, sex, drinking, hypertension, diabetes, gout, working posture, exercising hours per week, education level, osteophytes, Ca^2+^, and lumbar puncture, were selected as potential predictors (Figure 2). Finally, the results in both the univariate logistics regression and LASSO regression analysis were used for multivariable logistic regression analysis and revealed seven risk factors with *p* < 0.1, namely, age, sex, hypertension, diabetes, gout, working posture, and exercising hours per week (Table 3).

### 3.3. Establishment and Evaluation of Nomogram for Predicting the Risk of IVDD

**Figure 3:**
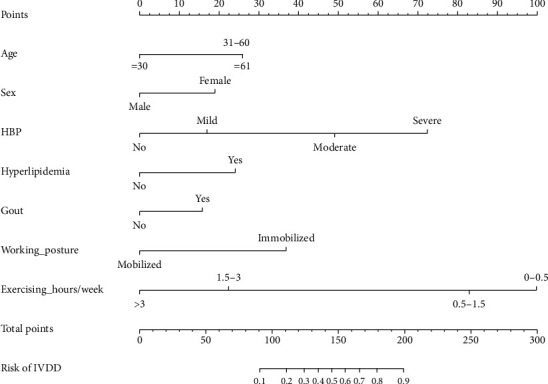
The IVDD nomogram. The predicting nomogram was developed with factors including age, sex, hypertension, diabetes, gout, working posture, and exercising hours per week.

**Figure 4:**
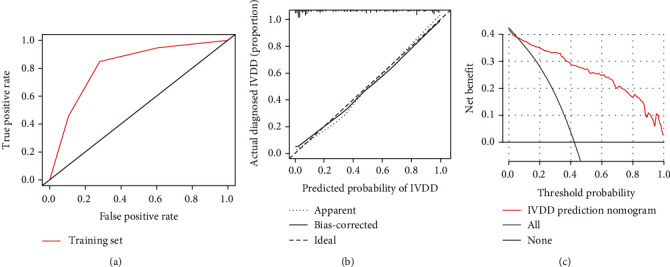
The ROC curve, calibration curve. and DCA curve of the training group. (a) The ROC curve of the nomogram model. (b) The calibration curve of the training group. (c) The decision curve of the training group.

The selected seven independent predictors above were used to establish the predictive nomogram (Figure 3). A comprehensive evaluation of the nomogram was carried out. The AUC of ROC curve of the nomogram model was 0.815 (95% CI, 0.759-0.870), indicating that the discrimination performance of the nomogram model was satisfactory (Figure 4(a)). Meanwhile, the favorable discrimination of the nomogram was confirmed by the *C*-index (0.916, (95% CI, 0.876-0.956)). In addition, the calibration plot revealed great agreement of the observed results and the predicted probability in this study (Figure 4(b)). To identify the clinical benefit of the predictive nomogram, the clinical practicability of it was also evaluated through DCA. The DCA (in a range of risk thresholds 0.01 to 1.00) indicated that the nomogram had a high net benefit (Figure 4(c)).

### 3.4. Validation of the Nomogram Prediction Model

**Figure 5:**
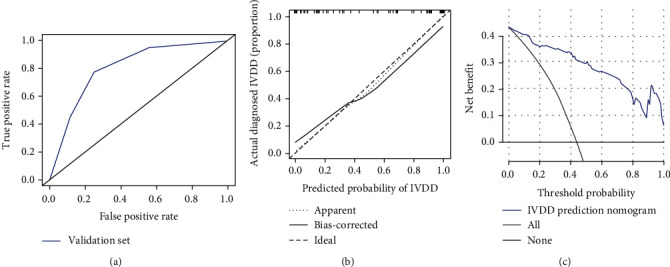
The ROC curve, calibration curve, and DCA curve of the validation group. (a) The ROC curve of the validation group. (b) The calibration curve of the validation group. (c) The decision curve of the validation group.

The data of the validation group were utilized to validate the nomogram above. In the validation cohort, the AUC of nomogram model was 0.805 (95% CI, 0.718-0.892) (Figure 5(a)) and the *C*-index of nomogram was 0.949 (95% CI, 0.909-0.989). Consistently, the calibration plot also indicated a great consistency with the results (Figure 5(b)). Furthermore, the DCA results demonstrated that when the nomogram was used to assess the validation set, the model also showed good net benefit (Figure 5(c)).

### 3.5. Exploring the Relationship between the Severity of IVDD and the AngII Level

**Figure 6:**
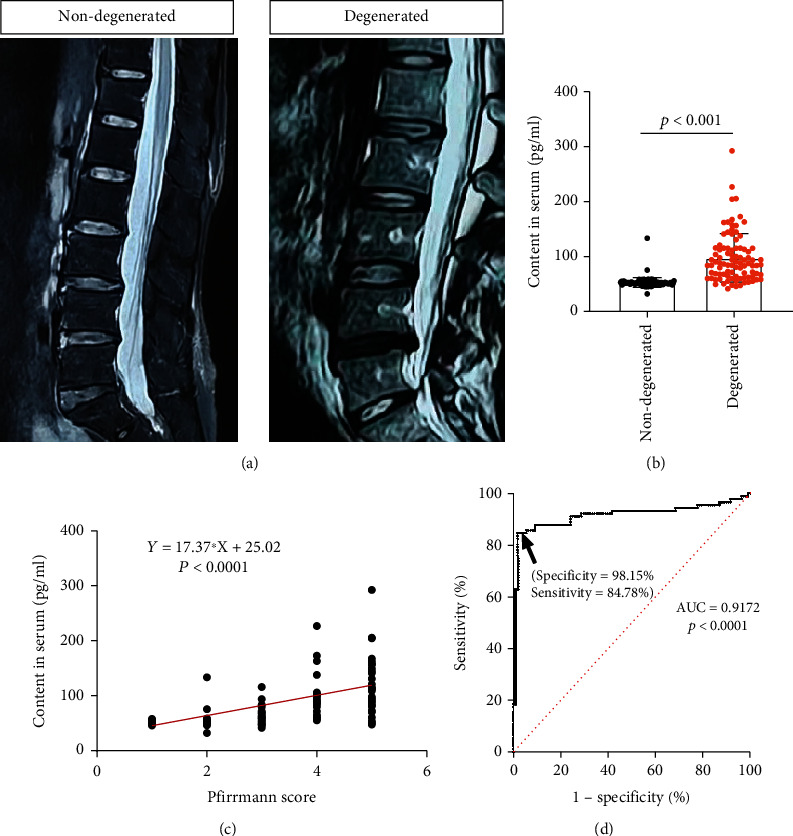
The relationship between the severity of IVDD and the AngII level in the peripheral blood. (a) The MRI results of non-degenerated patients and degenerated patients. (b) The level of AngII in peripheral blood of patients in the two groups. (c) Correlation analysis between AngII level in peripheral blood and the severity of IVDD. (d) The ROC curve analysis was used to test the ability of the AngII level to predict the risk of IVDD.

The AngII plays a pivotal role in the RAS. Aberrant activation of the RAS system can increase the level of AngII in the peripheral blood, which may enter the degenerated discs after vascular ingrowth. Based on the above results indicating the close relationship between IVDD and hypertension, we wonder whether IVDD and AngII are independently related. Our previous study found that AngII can lead to the dysfunction of nucleus pulposus cells. To explore whether AngII is an independent risk factor for IVDD, the peripheral blood samples of 108 patients with IVDD and of 92 university students without IVDD were collected (Figure 6(a)). Characteristics of the participants (108 patients and 92 students) were summarized in table 4. Despite significant difference in the age, no significant difference was observed in any other parameters. And the AngII levels in the peripheral blood of patients with various Pfirrmann grades were detected through ELISA. The results revealed that the average level of AngII in the nondegenerated group was 52.91 ± 9.01 pg/mL, whereas the average AngII level in the degenerated group (97.62 ± 44.02 pg/mL) was significantly higher (*p* < 0.001) (Figure 6(b)). In addition, we explored the relationship between the severity of IVDD and the AngII level. The results of Pearson's correlation revealed that the severity of IVDD was closely associated with the AngII level. The severity of IVDD was correlated with AngII level (Pearson's *R*^2^ = 0.4478, *p* < 0.001) (Figure 6(c)). Additionally, the AUC of the ROC curve was 0.9172 (*p* < 0.001), indicating that the AngII level in the peripheral blood was an excellent predictor of IVDD (Figure 6(d)). The above results revealed that the AngII level was closely associated with the severity of IVDD, indicating that the AngII in the peripheral blood could be applied as the auxiliary diagnostic index of IVDD.

## 4. Discussion

Although there are complex and multifactorial causes for the development of LBP, it is widely accepted that IVDD is the major contributor to LBP [19]. IVDD can be affected by both genetic and environmental factors. However, environmental factors, such as lifestyles and dietary habits that are closely relevant to metabolic diseases and systemic diseases, can be consciously controlled and modified to improve human's health. Adverse lifestyle-related factors including high BMI, lack of physical activity and smoking can lead to or aggregate IVDD [20]. In addition, previous studies reported that metabolic diseases such as diabetes and obesity were associated with the development of IVDD [6]. In addition, metabolic disease is a kind of systemic disease that can affect many organ systems including intervertebral disc [21]. All these risk factors above should be emphasized in the prevention and control of IVDD.

To prevent and curb the development of IVDD, it is essential to establish a model to predict the potential risk factors. Several blood lipid-metabolism-related genes were previously selected as candidate predictive biomarkers for IVDD [15]. However, a comprehensive and useful predictive model for IVDD is still lacking. In this present study, based on potential risk factors, a predictive nomogram for IVDD was established. A total of 309 patients were included in this study, and seven independent predictors including age, sex, hypertension, diabetes, gout, working posture, and exercising hours per week were selected to construct the predictive nomogram. The AUC of the model was 0.815 (95% CI, 0.759-0.870), which indicated good discrimination ability. Hypertension and the weight of exercising hours per week were the highest power factors shown in the nomogram, followed by working posture, diabetes, gout, age, and sex.

Hypertension is one of the major chronic metabolic diseases. It has long been known that the renin-angiotensin system (RAS) is essential in the development of hypertension [22]. In addition, local tissue RAS (tRAS) was observed in many tissues such as the brain, kidney, pancreas, and adipose tissue [23]. Local tRAS is not only associated with hypertension but also relevant to degeneration- or inflammation-related diseases in many tissues. Our previous study revealed that the activation of tRAS in the nucleus pulpous tissues was significantly associated with the development of IVDD [8]. In addition, we revealed that AngII could induce the degeneration and fibrosis of NPCs [8]. To verify the role of AngII in the development of IVDD, the spontaneously hypertensive rat (SHR) was used in our previous study [8]. The results revealed that the local tRAS was also activated in SHR nucleus pulposus tissue, accompanied by higher level of angiotensin-converting enzyme (ACE). In addition, the results of immunofluorescence demonstrated higher level of MMP 3 and lower level of collagen type II in SHR nucleus pulposus tissue. The above findings in previous studies indicated that hypertension may correlate with IVDD. Consistently, the nomogram of this present study indicated that individuals with hypertension tend to have greater points and the higher the blood pressure is, the higher the points for predicting IVDD. Alternatively, previous studies reported that patients with osteoarthritis, a similar disease to IVDD, tend to have relatively higher risk of cardiovascular diseases including hypertension than those without osteoarthritis [24]. In a meta-analysis, Lo et al. also pointed that hypertension resulted in a 62% increase in knee osteoarthritis [25]. In addition, the results of the meta-analysis revealed that hypertension is a harmful factor rather than protective factor in the development of osteoarthritis [25]. Interestingly, a growing number of researches revealed a close relationship between metabolic diseases and osteoarthritis [26, 27]. Because of physiological similarities between the articular cartilage and the intervertebral disc cartilage [28], it may be plausible to assume that hypertension is one of the risk factors for IVDD Consistently, this present study indicated that the *p* value of both diabetes and hypertension was less than 0.05 in the multivariate logistic regression analysis, indicating that diabetes was another potential risk factor of IVDD. Zheng et al. reported that in the condition of diabetes, human islet amyloid polypeptide (hIAPP) oligomers can promote the expression of IL-1*β* that is responsible for intervertebral disc degeneration [29]. Besides, Russo et al. reported that diabetes can lead to IVDD through promoting ECM degradation and cell apoptosis. The results of our study are consistent with previously reported findings. Although both hypertension and diabetes are systemic metabolic diseases, the results in the present study showed that patients with moderate or severe hypertension were at relatively higher risk of IVDD than patients with diabetes. More studies are still required to validate the exact effects of metabolic diseases on IVDD.

AngII is the major mediator to hypertension [30]. A previous study reported that AngII can result in a series of pathological changes including oxidative stress, inflammation, and fibrosis [31]. Therefore, we want to explore whether AngII is an independent risk factor for IVDD. The peripheral blood samples of participants with or without IVDD but not hypertension were collected. Our prospective exploration results revealed that the mean AngII level in the peripheral blood of patients with IVDD was remarkably higher than that of individuals without IVDD. Pearson's correlation analysis indicated a remarkable correlation between AngII level and the severity of IVDD. And the AUC of the ROC curve was 0.9172, indicating that AngII level showed a good predictive power for IVDD. In the prospective exploration, nonhypertensive patients were included for two main purposes: first is to rule out medication effect; second is to seek out hypertension-independent risk factors. The results of the study suggested that AngII may be developed to be a potential predictive factor for IVDD.

Physical activity plays an important role in maintaining human's body health. Liu et al. reported that exercises can increase endurance and muscle strength [32]. The muscles can provide support to the spine, which can be strengthened through exercises. However, lack of exercises can result in poor physical condition with decreased muscle strength. Iki et al. reported that regular exercise is beneficial for bone health [33]. In their study, patients with poor trunk muscle strength and lack of exercise showed greater risk for bone loss. In contrast, participants who have regular exercises tend to be at relatively lower risk of osteoporosis. It is widely accepted that the decreased bone mineral density can negatively affect the biomechanics of the spinal column, which subsequently induce the damage to the nucleus pulposus and annulus fibrosus [34]. In this present study, the exercising hours per week was graded into four categories: ≤0.5 hours, >0.5 and ≤1.5 hours, >1.5 and ≤3 hours, and >3 hours. The results uncovered that individuals who spent less time in exercises tended to have higher points in the nomogram and people with more than 3 hours of exercises every week had the least points, indicating that regular exercises can help lower the risk of IVDD. This may be because exercise can promote blood circulation to the intervertebral disc and strengthen the endurance and flexibility of the spinal muscle that helps maintain the spine stability and protect against IVDD. As for the immobilized working posture, one of the risk factors in the nomogram, Park et al. reported that adversely fixed working postures of dentists can cause musculoskeletal diseases especially in the lower back and neck [35]. This was in line with our results. As a result, regular exercises may help delay or attenuate the incidence of IVDD.

Moreover, other factors in the nomogram, which included old age, female gender, and gout, were also potential risk factors of IVDD. IVDD is an ageing-related disease. With the increase in age, the number of the nucleus pulposus cells and the content of water and ECM in the intervertebral disc will be reduced. This can impair the ability of intervertebral disc to absorb shock and stress, which might aggravate the process of IVDD. The results of the nomogram showed that women had higher risk in the risk evaluation of IVDD than men. Despite the protective effects of estrogen on musculoskeletal structures such as IVD [21], the high morbidity of IVDD in women may be due to pregnancy, menopause, and relatively weak spinal muscular strength. Although it was reported that IVDD was relevant to high BMI [20], the results of the present study showed that BMI was not statistically significant in univariate logistics regression analysis and not the strongest predictors in the LASSO regression analysis. Gout as a metabolic disease was also one of the potential risk factors in the development of IVDD. Patients with gout were reported with the accumulation of urate crystals in the bone joints, kidneys, and subcutaneous sites [36]. The results of this study revealed that IVDD was associated with gout. We speculated that this may be because urate crystal deposits in the IVD and its surrounding structures such as muscles and ligaments.

Based on the potential risk factors for IVDD, a prediction nomogram model was developed. The accuracy of the prediction model was assessed by various methods, and the results showed that this model could effectively predict the risk of IVDD. In addition, based on the model, AngII was revealed to be a possible diagnostic indicator of the IVDD.

### 4.1. Limitations

There are also several limitations in the present study. First and foremost, patients with severe metabolic diseases, such as hypertension, tend to be treated with medication, but the effects of medication on IVDD were not considered in the study. Further studies about the direct or indirect effects of medication on the process of IVDD are still required. Besides, biased results could be caused by a limited sample size. In addition, cases were collected over a short period of time and a relatively geographic area. Hence, a multicenter prospective study with a larger sample size is required to investigate other factors. What is more, majority of hypertensive patients require various kinds of RAS antagonists to control the blood pressure. But the present study cannot prove that RAS antagonists can attenuate IVDD or reduce the frequency of IVDD-related surgeries. Future research needs to be carried out from the perspective of cardiology. This can be confirmed through longitudinal, large sample size, cohort studies. Finally, in this study, 92 individuals without IVDD were relatively younger than those with IVDD. In a future study, the comparison between age-matched groups should be performed using a pair-sample *t*-test because age is also a potential risk factor for IVDD.

## Figures and Tables

**Table 1:** Characteristics of participants in the training group and the validation group.

Variables	No. (%)		*χ* ^2^	*p* value
	Training group (217)	Validation group (92)		
IVDD				
No	125 (57.60)	52 (56.52)	0.031	0.86
Yes	92 (42.40)	40 (43.48)		
Age, year				
≤30	71 (32.72)	27 (29.35)	2.184	0.336
>30 and ≤60	82 (37.79)	30 (32.61)		
>60	64 (29.49)	35 (38.04)		
Sex				
Male	115 (53.00)	42 (45.65)	1.394	0.238
Female	102 (47.00)	50 (54.35)		
Smoking				
No	117 (53.92)	51 (55.43)	0.060	0.807
Yes	100 (46.08)	41 (44.57)		
Drinking				
No	122 (56.22)	49 (53.26)	0.229	0.632
Yes	95 (43.78)	43 (46.74)		
BMI				
<18.5	36 (16.59)	13 (14.13)	5.985	0.112
≥18.5 and<24	63 (29.03)	35 (38.04)		
≥24 and <28	75 (34.56)	35 (38.04)		
≥28	43 (19.82)	9 (9.78)		
Hypertension				
No	148 (68.20)	70 (76.09)	3.712	0.294
Mild	24 (11.06)	5 (5.43)		
Moderate	18 (8.29)	9 (9.78)		
Severe	27 (12.44)	8 (8.70)		
Diabetes				
No	162 (74.65)	62 (67.39)	1.709	0.191
Yes	55 (25.35)	30 (32.61)		
Gout				
No	172 (79.26)	74 (80.43)	0.055	0.815
Yes	45 (20.74)	18 (19.57)		
Marital status				
No	111 (51.15)	46 (50.00)	0.034	0.853
Yes	106 (48.85)	46 (50.00)		
Working posture				
Immobilized	138 (63.59)	62 (67.39)	0.408	0.523
Mobilized	79 (36.41)	30 (32.61)		
Exercising hours/week				
≤0.5	55 (25.35)	24 (26.09)	0.897	0.826
	58 (26.73)	20 (21.74)		
>1.5 and ≤3	49 (22.58)	23 (25.00)		
>3	55 (25.35)	25 (27.17)		
Education level				
High school	82 (37.79)	31 (33.70)	1.606	0.448
Bachelor degree	99 (45.62)	49 (53.26)		
Graduate degree	36 (16.59)	12 (13.04)		
Modic changes				
No	153 (70.51)	66 (71.74)	0.048	0.827
Yes	64 (29.49)	26 (28.26)		
Osteophytes				
No	152 (70.05)	63 (68.48)	0.075	0.784
Yes	65 (29.95)	29 (31.52)		
Ca^2+^				
Normal	153 (70.51)	67 (72.83)	0.269	0.874
Low	36 (16.59)	15 (16.30)		
High	28 (12.9)	10 (10.87)		
Hb				
Normal	171 (78.80)	69 (75.00)	3.288	0.193
Low	33 (15.21)	12 (13.04)		
High	13 (5.99)	11 (11.96)		
Lumbar puncture				
No	166 (76.50)	71 (77.17)	0.017	0.898
Yes	51 (23.50)	21 (22.83)		

**Table 2:** Univariate logistic regression analysis of the training set.

Variables	IVDD (*n* = 92)	Non-IVDD (*n* = 125)	*χ* ^2^/*Z*	*P* value
Age (year)				
≤30	20 (21.74)	51 (40.80)	-3.548	<0.001
>30 and ≤60	37 (40.22)	45 (36.00)	2.147	0.032
>60	35 (38.04)	29 (23.20)	3.086	0.002
Sex				
Male	49 (53.26)	66 (52.80)	-1.579	0.114
Female	43 (46.74)	59 (47.20)	-0.067	0.946
Smoking				
No	44 (47.83)	61 (48.80)	-1.931	0.054
Yes	48 (52.17)	64 (51.20)	0.442	0.659
Drinking				
No	52 (56.52)	60 (48.00)	-1.265	0.206
Yes	40 (43.48)	65 (52.00)	-0.630	0.529
BMI				
<18.5	17 (18.48)	19 (15.20)	-0.333	0.739
≥18.5 and <24	23 (25.00)	40 (32.00)	-1.042	0.297
≥24 and <28	33 (35.87)	42 (33.60)	-0.319	0.749
≥28	19 (20.65)	24 (19.20)	-0.270	0.787
Hypertension				
No	48 (52.17)	100 (80.00)	-4.18	<0.001
Mild	9 (9.78)	15 (12.00)	0.489	0.625
Moderate	12 (13.04)	6 (4.80)	2.693	0.007
Severe	23 (25.00)	4 (3.20)	4.360	<0.001
Diabetes				
No	55 (59.78)	107 (85.60)	-4.011	<0.001
Yes	37 (40.22)	18 (14.40)	4.177	<0.001
Gout				
No	71 (77.17)	101 (80.80)	-2.276	0.023
Yes	21 (22.83)	24 (19.20)	0.650	0.515
Marital status				
No	47 (51.09)	64 (51.20)	-1.607	0.108
Yes	45 (48.91)	61 (48.80)	0.016	0.987
Working posture				
Immobilized	75 (81.52)	63 (50.40)	1.020	0.308
Mobilized	17 (18.48)	62 (49.60)	-4.549	<0.001
Exercising hours/week				
≤0.5	42 (45.65)	13 (10.40)	3.695	<0.001
>0.5 and ≤1.5	36 (39.13)	22 (17.60)	-1.631	0.103
>1.5 and ≤3	9 (9.78)	40 (32.00)	-5.475	<0.001
>3	5 (5.43)	50 (40.00)	-6.137	<0.001
Education level				
High school	31 (33.7)	51 (40.80)	-2.186	0.029
Bachelor degree	46 (50.00)	53 (42.40)	1.171	0.242
Graduate degree	15 (16.30)	21 (16.80)	0.396	0.692
Modic changes				
No	67 (72.83)	86 (68.80)	-1.532	0.126
Yes	25 (27.17)	39 (31.20)	-0.642	0.521
Osteophytes				
No	64 (69.57)	88 (70.40)	-1.938	0.053
Yes	28 (30.43)	37 (29.60)	0.133	0.895
Ca^2+^				
Normal	64 (69.57)	89 (71.20)	-2.012	0.044
Low	14 (15.22)	22 (17.60)	-0.322	0.747
High	14 (15.22)	14 (11.20)	0.800	0.424
Hb				
Normal	73 (79.35)	98 (78.40)	-1.905	0.057
Low	14 (15.22)	19 (15.20)	-0.028	0.977
High	5 (5.43)	8 (6.40)	-0.297	0.766
Lumbar puncture				
No	75 (81.52)	91 (72.80)	-1.240	0.215
Yes	17 (18.48)	34 (27.20)	-1.490	0.136

**Table 3:** Multivariable logistic regression analysis of the training group.

Intercept and variable		Prediction model	
	*β*	OR (95% CI)	*p* value
Intercept	-0.157	0.855 (0.201-3.555)	0.829
Age			
≤30			
>30 and ≤60	0.876	2.402 (0.878-6.843)	0.092
>60	1.029	2.798 (0.953-8.656)	0.065
Sex			
Male			
Female	0.873	2.394 (0.971-6.231)	0.064
Smoking			
No			
Yes	-0.276	0.759 (0.296-1.887)	0.556
Drinking			
No			
Yes	-0.448	0.639 (0.243-1.627)	0.351
Hypertension			
No			
Mild	1.111	3.038 (0.772-12.407)	0.113
Moderate	1.842	6.309 (1.466-32.023)	0.018
Severe	3.587	36.113 (5.765-346.302)	<0.001
Diabetes			
No			
Yes	1.085	2.960 (1.045-8.918)	0.046
Gout			
No			
Yes	0.924	2.520 (0.902-7.395)	0.083
Working posture			
Immobilized			
Mobilized	-1.553	0.212 (0.080-0.517)	<0.001
Exercising hours/week			
≤0.5			
>0.5 and ≤1.5	-0.742	0.476 (0.160-1.354)	0.170
>1.5 and ≤3	-3.263	0.038 (0.009-0.134)	<0.001
>3	-4.305	0.013 (0.002-0.058)	<0.001
Education level			
High school			
Bachelor degree	0.434	1.543 (0.565-4.315)	0.399
Graduate degree	0.184	1.201 (0.394-3.669)	0.745
Osteophytes			
No			
Yes	-0.513	0.598 (0.229-1.517)	0.284
Ca2+			
Normal			
Low	-0.032	0.968 (0.275-3.354)	0.959
High	0.715	2.044 (0.596-7.368)	0.260
Hb			
Normal			
Low	-0.084	0.919 (0.290-2.816)	0.884
High	-0.340	0.711 (0.108-4.291)	0.713
Lumbar puncture			
No			
Yes	-0.078	0.925 (0.332-2.567)	0.880

**Table 4:** Characteristics of the participants involved in exploring the relationship between IVDD and AngII.

Variables	IVDD (*n* = 108)	Non-IVDD (*n* = 92)	*T* value	*p* value
Age	41.36 ± 13.44	28.97 ± 4.01	8.525	<0.001
Sex, female (%)	55 (50.93)	45 (48.91)	0.081	0.777
Smoking, yes (%)	52 (48.15)	45 (48.91)	0.012	0.914
Drinking, yes (%)	49 (45.37)	44 (47.83)	0.120	0.729
BMI	23.43 ± 4.26	23.13 ± 4.29	0.490	0.625

## Data Availability

The dataset of this article is available on request from the corresponding authors.

## Abbreviations

LBP: Low back pain
IVDD: Intervertebral disc degeneration
IVD: Intervertebral disc
LASSO: Least absolute shrinkage and selection operator regression
ROC: Receiver operating characteristic
DCA: Decision curve analysis
AUC: Area under the curve
tRAS: Tissue renin-angiotensin system
RAS: Renin-angiotensin system
CT: Computed tomography
MRI: Magnetic resonance imaging
SHR: Spontaneously hypertensive rat
NP: Nucleus pulpous
AngII: Angiotensin II.
